# Clinical Impact of a Digital Dose Counter Pressurized Metered-Dose Inhaler on Uncontrolled Asthma: Cross-Sectional, Observational, Surveillance Study

**DOI:** 10.2196/13530

**Published:** 2019-05-05

**Authors:** Randeep Guleria, Krishnaprasad Korukonda, DUSS Investigators

**Affiliations:** 1 All India Institute of Medical Sciences New Delhi India; 2 Chembur Clinic Mumbai India; 3 DUSS panel India

**Keywords:** asthma, drug utilization surveillance, pseudoadherence, xanthines, anti-asthmatic agents

## Abstract

**Background:**

In India, control of asthma with persistent symptoms remains a clinical enigma with likely incriminating factors including non- and pseudoadherence to the inhaled corticosteroids and long-acting beta2-agonists. The United States Food and Drug Administration guidance recommends the use of dose counter pressurized metered-dose inhalers (pMDIs) with further mechanisms to track adherence and pseudoadherence in real-world settings.

**Objective:**

Digital dose counter pMDIs (dpMDIs) offer simplified, reliable tracking of individual “actuated” dosages with “END” display at completion of the labelled therapeutic aerosol spray. The translational impact on symptom persistence with likely unwarranted exposure to the “Step up” strategy is often prevented if not treated, as in the cases of “pseudo” severe asthma. To further assess the real-world acceptance and clinical impact of dpMDIs in bronchial asthma including poorly controlled or uncontrolled bronchial asthma cases, a noninterventional observational study was performed.

**Methods:**

This cross-sectional, retrospective, case cohort, observational study—the Drug Utilization Surveillance—of dpMDIs in bronchial asthma was conducted in September 2016 in an outpatient setting in India. The retrospective analysis was initiated and conducted as per the International Conference on Harmonization Good Clinical Practice principles and Declaration of Helsinki, following approval from the local ethics committee and registration in the Clinical Trial Registry of India.

**Results:**

Consecutive cases of moderate-to-severe asthma with poor control (n=4575), diagnosed as per the Global Initiative for Asthma symptom scale at baseline and follow-up, were included. Patients under treatment using dpMDIs were enrolled from 500 centers across India and assessed by respiratory care specialists. Baseline asthma control was assessed as partly controlled (n=4575) or poorly controlled (n=2942). Per protocol analyses showed that asthma was well controlled with dpMDIs at 8 weeks in 92.7% of the cases (2727/2942, *P*<.001). Adverse events (n=106, 2%) of mild-to-moderate intensity were reported. Nebulization was required in two patients with episodic breathlessness who were discharged with no consequent sequelae. Post hoc analyses for patients with baseline poorly controlled asthma who “switched” exclusively to dpMDI monotherapy or a combination with xanthines or long-acting beta2-agonists showed a “well controlled” asthma status in 85.9% (500/582, *P*=.04), 95.4% (395/414, *P*=.048), and 80.3% (106/132, *P*=.28) of the cases, respectively. The patient acceptability criteria for an “empty” canister was well correlated with the clinical strategy to identify and avoid pseudoadherence in poorly controlled or difficult-to-treat asthma cases, especially in patients who “switched” exclusively to dpMDIs (n=582) and demonstrated responses of “Use till twenty dose display” (65/156, 41.6%), “Use till END display” (83/156, 53.2%), and “Use till LAST spray” (8/156, 5.1%).

**Conclusions:**

dpMDIs offer simple, accurate, and reliable tracking of non- and pseudoadherence while highlighting incremental asthma-control rates in severe and pseudosevere asthma cases before risk assessment for further “add-on” therapy

**Trial Registration:**

Clinical Trials Registry - India CTRI/2018/06/014595; http://www.ctri.nic.in/Clinicaltrials/pmaindet2.php? trialid=24583

## Introduction

Bronchial asthma continues to be a serious global health challenge with an estimated global prevalence of 300 million people, where most cases remain poorly controlled [1-3].

### Problem Statement

Patient-reported asthma control worldwide, especially in India and China, remains remarkably low, varying from 0% to 50% [4,5]. The correlation of the patient-reported satisfaction scores and the Global Initiative for Asthma (GINA) symptom scale for asthma control reveals disproportionate or alarming rates of partly controlled (48%-60%) or uncontrolled (18%) asthma, especially in patients receiving background inhaled corticosteroid and long-acting beta2-agonist (ICS/LABA) combination inhaler therapies [4,6,7]. Asthma control is a complex and multiparametric issue that is largely affected by not only physiological and environmental parameters, but also the psychological state of patients and their cultural and socioeconomic background [8]. In the Indian subcontinent, the clinical issues of patient-reported social stigma, habituation, tolerance, and adverse events with inhaler therapies are often perceived to be complex and perplexing for primary care physicians.

Despite the enumeration of these likely confounding variables or concomitant risk factors, asthma control continues to remain an elusive goal, regardless of the availability of new devices or therapeutic strategies including biologics or anti-Ig E therapy [9-12].

The pressurized metered-dose inhaler (pMDI) has been the most widely used inhaler over the past 40 years and a value-added proposition, unlike the dry-powder inhaler. The pMDI offers incremental lung-deposition rates, especially in patients with severe asthma or poor inspiratory flow rates. However, in most cases, they provide little information on the “remaining” medication or therapeutic dosages in the canister [13]. This uncertainty leads to pMDI overuse beyond the stated labelled number of dosages, and the patient inhales little or “tail” sprays containing only propellant. The clinical implications of this are huge, with the patients often dealing with persistent symptoms or exacerbations requiring further investigations or unwarranted approach with advanced therapies or bronchodilators. Ogren found that up to 40% of patients believe they are taking their asthma medication when they are actually activating an empty or nearly empty pMDI [14]. Similarly, an epidemiological survey (B Singh, MD, personal communication, 2016) conducted across India seconded the viewpoint of most pulmonologists in highlighting patients’ persistence with the conventional pMDIs till the “last” spray, creating a false perception that they are actually receiving medication, which leads to pseudoadherence and related complications [15].

### International Recommendations

The United States Food and Drug Administration took cognizance of these twin challenges of non- and pseudoadherence, especially with pMDI devices used to deliver ICS/LABA combinations, and recommends the use of dose counter pMDIs to track nonadherence accurately and reliably through individual dosage movement or actuation, with “lockout” mechanisms to avoid inhalation of the tailed sprays [16]. However, the use of “lockout” mechanisms may not be clinically relevant when delivering formulations with dual use such as maintenance and rescue medications.

Digital dose counter pMDIs (dpMDIs) offer simplified, reliable tracking of individual “actuated” dosages with END display that signifies the onset of pseudoadherence or “empty” sprays containing propellant only. The translational impact on symptom persistence with likely unwarranted exposure to the “Step up” strategy is often prevented if not treated, as in the cases of “pseudo” severe asthma (ie, Step 3 Asthma control cases receiving Step 4 care).

### Objective

In line with the global and local epidemiological burden of uncontrolled or partly controlled asthma, which varies from 58% to 60% for patients on the current standard of care [4,7], the real-world utilization and impact of dpMDI initiation or “switch” was evaluated in this retrospective, observational, drug-utilization clinical study.

## Methods

This cross-sectional, retrospective, case cohort analysis—the Drug Utilization Surveillance study (Clinical Trials Registry - India CTRI/2018/06/014595)—of dpMDIs was performed in patients with bronchial asthma after obtaining approval from the local ethics committee, with registration in the Clinical Trial Registry of India. Patients were enrolled from 500 outpatient centers that utilized the GINA symptom scale for assessing asthma control in patients on dpMDIs across India in September 2016. The study was conducted as per the principles of International Conference of Harmonization for Good clinical practice and Declaration of Helsinki while ensuring confidentiality of patient identifiers before analyses.

Consecutive case records for patients with bronchial asthma exposure to dpMDIs were collated for analyses with follow-up information on the asthma control status for at least 8 weeks. Primary analyses for clinical cases were performed to assess the asthma control status with symptomatic assessment using the GINA symptom scale for daytime symptoms, night-time symptoms, activity limitation, and use of rescue medications for at least 8 weeks with dpMDIs. As per the GINA scale, asthma control was defined as well controlled, partly controlled, or uncontrolled, with total scores of 0, 1-2, and 3-4, respectively, at baseline, 4 weeks, and 8 weeks (follow-up). Clinician assessment or review of the inhalation technique, including patient feedback on the use of dpMDIs at 4 weeks, was also analyzed.

### Efficacy Parameters

Primary analysis, post hoc analyses, and interaction tests were performed for well-controlled asthma overall, in newly diagnosed cases, and in poorly controlled asthma cases at baseline, respectively. Statistical analyses for categorical and numerical data were carried out by the Fisher exact test and Student *t* test, using QuickCalcs GraphPad Prism (version 7.05; San Diego, CA), with two-tailed *P* values<.05 considered statistically significant.

### Safety Parameters

Descriptive statistics were used for assessment of the incidence of treatment-emergent adverse events at 8 weeks.

## Results

A total of 4575 consecutive cases of moderate-to-severe asthma with uncontrolled status, as assessed by respiratory care specialists, of at least one GINA symptom at baseline and follow-up were enrolled. Patients included were under treatment with dpMDIs from 500 centers across India. Asthma control status was categorized as partly controlled (n=4575) or uncontrolled (n=2942) at baseline. Per protocol analyses were performed for patients with uncontrolled asthma, as evaluated by the GINA symptom scale at 8 weeks. Baseline demographics are presented in Table 1. More patients were on formoterol/budesonide combination treatment (n=3791, 73%) than on salmeterol/fluticasone combination treatment (n=1404, 27%).

**Figure 1:**
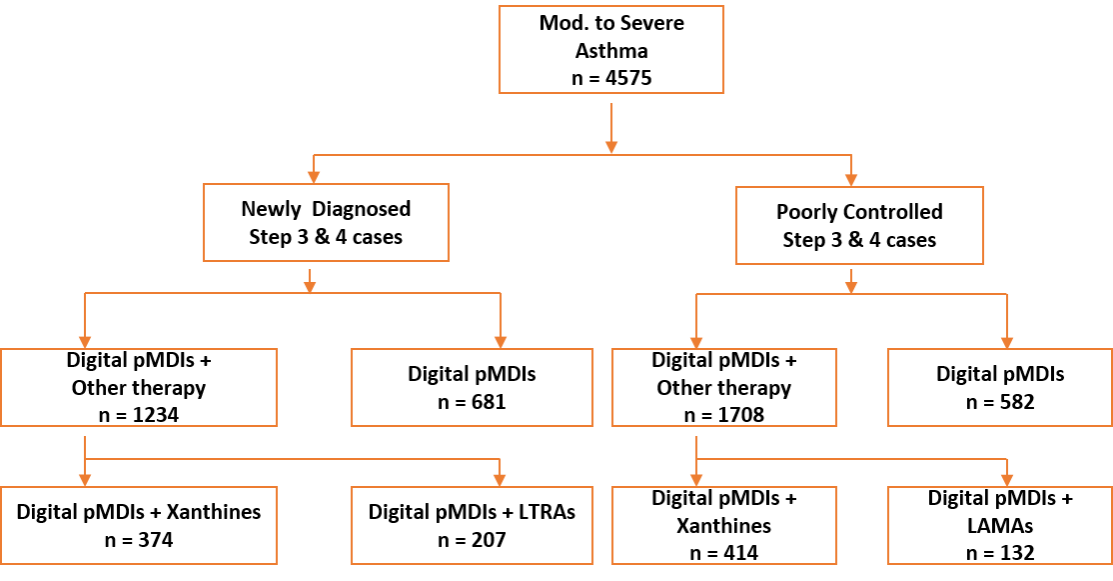
Patient disposition flow chart to digital dose counter pMDIs at baseline. LTRAs: Leukotriene receptor antagonists, LAMAs: Long-acting muscarinic antagonists, pMDIs: pressurized metered dose inhalers.

Clinical records were available for 4575 cases, with further per protocol analyses conducted for patients with at least two follow-up visits (n=2942, uncontrolled asthma status at baseline), as highlighted in the patient disposition chart (Figure 1).

Asthma control status at 4 and 8 weeks was categorized as well controlled, partly controlled, or uncontrolled as per the available GINA assessment total scores for daytime, activity limitation, night-time symptoms, and use of rescue medications in the last month. The baseline and follow-up symptoms were assessed as uncontrolled, partly controlled, or well controlled based on the GINA assessment scale for symptom and rescue medication use (Table 2).

**Table 1:** Demographics and clinical characteristics at baseline.

Parameter			Value
**Asthma control, n (%)**			
	Partly controlled		4575 (100)
	Uncontrolled		2942 (64.3)
**Characteristics of patients with uncontrolled asthma**			
	**Gender, n (%)**		
		Male	2114 (71.9)
		Female	828 (28.1)
	Age, mean (SD)		49.5 (15.9)
	Body weight, mean (SD)		62.8 (13.2)
	Newly diagnosed, n (%)		1234 (41.9)
	Poorly controlled, n (%)		1708 (58.1)
	Exacerbation history, n (%)		135 (4.6)
**Baseline medications, n (%)**			
	Antibiotics		24 (0.8)
	Bronchodilator syrup		200 (6.8)
	Oral steroids		218 (7.4)
	Inhaled corticosteroids/long-acting β2 agonist		1194 (43.9)
	Xanthines		110 (4.0)
	Leukotriene receptor anatagonist with or without antihistaminic agents		55 (2)
	Other combination		1359 (50)
	No therapy		514 (17.5)

**Table 2:** Patient symptom assessment scores for well-controlled, partly controlled, or uncontrolled asthma at every visit. The scores are marked as Yes (1) and No (0). Total scores of 3-4, 1-2, and 0 at each visit indicate uncontrolled, partly controlled, and well-controlled asthma, respectively.

Global Initiative for Asthma symptom scale	Score
Daytime asthma symptoms >2 times/week	1/0
Activity or exercise limited by asthma	1/0
Waking at night due to asthma	1/0
Rescue medication (number of times/month)	1/0

### Efficacy

Primary analyses based on clinical assessment for daytime, night-time, activity limitation symptomatology showed that a “well-controlled” status was observed in 92.7% cases (2701/2942, *P*<.001), 95.9% (1184/1234, *P*<.001), and 90.3% (1563/1708, *P*<.001) of the overall cases, newly diagnosed cases, or poorly controlled cases at baseline, respectively (Table 3).

Clinician assessment of patient acceptability and use of dpMDIs at 4 week was categorized as “Use till twenty dose display” (n=430, 33.4%), “Use till END display” (n=765, 59.4%), and “Use till LAST spray” (n=92, 7.3%).

Post hoc analyses for newly or poorly controlled cases receiving dpMDIs with or without concomitant therapy were further performed for patients with well-controlled asthma.

Well-controlled asthma was observed in 97.6% (365/374, *P*=.01) and 97.6% (202/207, *P<*.001) of patients with newly diagnosed asthma receiving xanthine and LTRAs, respectively.

Similarly, in the poorly controlled group at baseline, who “switched” exclusively to dpMDIs, the clinical response rates for well-controlled asthma was significantly high at 85.9% (500/582, *P*=.04) at 8 weeks. In the baseline poorly controlled group receiving dpMDIs with xanthines or long-acting muscarinic antagonist (LAMAs), the response rates for well-controlled asthma at 8 weeks were significant at 95.4% (395/414, *P*=.048) and 80.3% (106/132, *P*=.28), respectively.

Assessment of the “switch” group for dpMDIs by the health care specialists at 4 weeks showed the following responses: “Use till twenty dose display” (n=65, 41.6%), “Use till END display” (n=83, 53.2%), and “Use till LAST spray” (n=8, 5.1%).

### Safety Outcomes

Adverse events (n=106, 2%) of mild-to-moderate intensity involving tremors (n=34, 0.7%), palpitation (n=10, 0.2%), mouth ulcers (n=10, 0.2%), and oral candidiasis (n=9, 0.2%) were reported. Nebulization was required in two patients with episodic breathlessness, who were discharged with no consequent sequelae.

**Table 3:** Well-controlled asthma status at 8 weeks overall, in the newly diagnosed group, in the poorly controlled group, and in the uncontrolled group at baseline.

Patient population		Bronchial asthma cases, n (%)	Well-controlled asthma at 8 weeks, n (%)	*P* value
Baseline partly controlled asthma (overall) — dpMDI^a^/combination		4575 (100)	3955 (86.4)	<.001
Baseline uncontrolled asthma — dpMDI/combination		2942 (64.3)	2701 (92.7)	<.001
**Baseline newly diagnosed**				
	dpMDI/combination	1234 (58)	1184 (95.9)	<.001
	dpMDI/xanthine	374 (17.6)	365 (97.6)	.01
	dpMDI/LTRA^b^	207 (9.7)	202 (97.6)	.001
**Baseline poorly controlled**				
	dpMDI/combination	1708 (69.8)	1563 (90.3)	<.001
	dpMDI/xanthine	414 (16.9)	395 (95.4)	.048
	dpMDI/LAMA^c^	132 (5.4)	106 (80.3)	.28
	dpMDI monotherapy	582 (23.8)	500 (85.9)	.04

^a^dpMDI: digital dose counter pressurized metered-dose inhalers.

^b^LTRA: leukotriene receptor antagonist.

^c^LAMA: long-acting muscarinic antagonist.

## Discussion

This real-world, cross-sectional, retrospective study highlights the clinical impact and utilization of dpMDIs in the management of uncontrolled asthma. The well controlled asthma rates of 92.7% (2727/2942, *P*<.001), 95.4% (394/414, *P*=.048), and 85.9% (500/582, *P*=.04) at 8 weeks for overall group and clinical cases who ‘switched’ from conventional pMDIs to dpMDIs with Xanthines or alone, respectively, provides the first data point on clinical response rates with any dpMDIs for asthma cases predominantly on ICS/LABA combination. There is sparse literature on similar studies; a postapproval, prescription event, clinical study conducted with analogue dose indicator pMDIs that included 13,464 patients on salmeterol/fluticasone (EVOHALER*) treatment for Reversible Obstructive Airway Disease reported a response rate of 62% [17].

Severe asthma remains largely uncontrolled when the differential diagnosis involves conditions that may mimic asthmatic symptoms (eg, extrathoracic hyperresponsiveness syndromes and vocal cord dysfunction) or comorbidities that may worsen disease control (eg, allergic or nonallergic rhinitis, chronic rhinosinusitis with or without nasal polyps, bronchiectasis, and gastroesophageal reflux); when the condition is investigated with little recognition or connection with possible incorrect inhaler techniques; or when treatment adherence is poor, including non- or pseudoadherence observed in most clinical cases, which often leads to severe or a difficult-to-control asthma state.

### Nonadherence and Difficult-to-Control Asthma

The REALISE (REcognise Asthma and LInk to Symptoms and Experience) Asia survey based on the GINA assessment scale suggested that the questionnaire for asthma control highlighted the disparity in the rates of the well-controlled status (only 53.2%), and most patients (86%) were nonadherent to maintenance therapy with aerosols, making them highly susceptible to developing persistent symptoms or progressive disease. Timely reminders or active counselling plays a critical role in improving or building treatment adherence for optimal control or prevention of exacerbations [18]. This was further highlighted by Krishnaprasad in a noninterventional, prospective, observational, single-arm study involving moderate-to-severe asthma cases requiring ICS/LABA inhalation. Telephonic monitoring conducted for 124 patients to assess asthma control utilizing the GINA assessment questionnaire highlighted consistency in the “well-controlled” asthma status (84%) at 1 year for treatment of adherent patients utilizing conventional pMDIs without dose counters [18-19]. However, in most real-world settings, outside the realms of controlled research framework, monitoring the patient for adherence remains a difficult proposition to assess and review, as suggested by the GINA.

### Pseudoadherence and Difficult-to-control Asthma

Our results and patient descriptors highlight the pertinent need for an inhalation device or strategy to address the likely behavioral or device-related risk factors that may be observed, such as old age, a history of noncompliance, socioeconomic variables, or cognitive deficits, for continued benefits especially in severe asthma or pseudo-severe asthma cases due to “intermittent” or pseudo-adherence when faced with responsive symptoms [20].

The clinical response or well-controlled asthma rate of 90.3% in the poorly controlled group on dpMDIs with other therapy shows significant credibility to the overall “therapeutic” impact of these devices in the real-world management of severe asthma before further prescribing “Step up” or Single Maintenance and Rescue Therapy, which are likely to expose patients to systemic side effects of high-dose inhaled corticosteroids.

Post hoc analyses for poorly controlled patients with Step 3 Asthma control who "switched" exclusively to dpMDI monotherapy showed a "well controlled" asthma status in 85.9% (500/582, P=.04), of the cases, respectively. This finding further consolidates the rationale of the therapeutic role of these devices for symptomatic or pseudo-severe asthma cases. This was seconded by the high rates of compliance and feedback by patients using the dpMDIs correctly till the END display, thereby avoiding the ill-effects of pseudoadherence.

The lack of a significant response to dpMDIs with LAMAs in this study may be precluded by the small sample size and a lack of investigation or understanding of the underlying pathophysiologic basis involving eosinophilic inflammation or fraction exhaled nitric oxide response scores for continued response to the background ICS/LABA combination.

### Study Limitations

The findings are limited by the retrospective nature of the analyses, which lacks a control for comparative assessment. However, to our knowledge, the results are the first to highlight the incremental or translational benefits of dpMDIs in patients with difficult-to-treat or pseudo-severe asthma, which is often treated with continued or high-dose inhaled corticosteroids.

### Conclusion

The dpMDIs offer a simple, accurate, and reliable solution to tracking non- and pseudoadherence in real-world settings, thereby preventing morbidity or mortality associated with such obstructive airway diseases. By tracking the effects of the use of both preventer (controller) and reliever (rescue) medications, the digital pMDI will engage and empower patients in their self-care, leading to improved adherence while enabling real-time monitoring of medication use and symptom flare-ups by caregivers and the health care community.

The dpMDIs also remain a clinically important, yet relevant strategy that delivers optimal responses in therapy-resistant severe asthma while endorsing the concept that they “treat” pseudo-severe asthma while preventing severe asthma management with biologics or xanthines.

## Abbreviations

dpMDI: digital dose counter pMDIs
LAMA: long-acting muscarinic antagonist
LTRA: leukotriene receptor antagonist
pMDI: pressurized metered-dose inhalers
REALISE: REcognise Asthma and LInk to Symptoms and Experience
